# Intravenous immunoglobulin remodels innate immune cell communication and induces differential autophagy pathways in Kawasaki disease

**DOI:** 10.3389/fimmu.2026.1753478

**Published:** 2026-02-25

**Authors:** Suraj Chandrabhan Singh, Sruthi Vijaya Retnakumar, Mrinmoy Das, Srini V Kaveri, Mano Joseph Mathew, Jagadeesh Bayry

**Affiliations:** 1 Department of Biological Sciences & Engineering, Indian Institute of Technology Palakkad, Palakkad, Kerala, India; 2 Institut National de la Santé et de la Recherche Médicale, Centre de Recherche des Cordeliers, Sorbonne Université, Université Paris Cité, Paris, France; 3 Division of Medical Research, SRM Medical College Hospital and Research Centre, SRM Institute of Science and Technology, Kattankulathur, Tamil Nadu, India; 4 EFREI Research Lab, Panthéon Assas University, Villejuif, France; 5 Laboratoire Génomique, Bioinformatique et Chimie Moléculaire, EA7528, Conservatoire National des Arts et Métiers, HESAM Université, Paris, France

**Keywords:** autophagy, innate immune cells, IVIG, Kawasaki disease, macroautophagy, LC3-II, pseudotime trajectory, selective autophagy

## Abstract

**Introduction:**

Intravenous immunoglobulin (IVIG), a therapeutic preparation of pooled normal IgG is extensively used as a first-line immunotherapy for many autoimmune and inflammatory diseases, including Kawasaki disease (KD). IVIG provides therapeutic benefits through several non-exclusive mechanisms. Our recent data demonstrate that IVIG induces autophagy in inflammatory innate immune cells, a finding further supported by observations in IVIG-treated myopathy patients. However, whether IVIG selectively activates specific autophagy pathways across distinct innate immune cell subsets remains unclear.

**Methods:**

Single-cell RNA sequencing data from peripheral blood mononuclear cells (PBMC) of healthy controls, acute untreated KD patients, and IVIG-treated KD patients were analysed. Differential gene expression, cell–cell communication, functional pathway enrichment of autophagy-related pathways, and pseudotime trajectory analyses were performed. Mechanistic studies were conducted in vitro using PBMC and monocytes from healthy donors treated with different IVIG preparations, Fc fragments, or inhibitors, followed by immunoblotting for LC3-II.

**Results:**

KD was characterized by expansion of inflammatory monocytes and low-density neutrophils with reduced NK and γδ T cells. IVIG therapy reshaped innate immune composition and partially restored coordinated immune networks. IVIG upregulated core macroautophagy genes across innate immune subsets, particularly ATG7 and UVRAG in monocytes. It also induced non-canonical LC3-associated phagocytosis and multiple selective autophagy pathways in a cell-type-specific manner. Pseudotime analysis revealed normalization of monocyte trajectories following treatment. IVIG-induced autophagy occurred independently of Fc fragments and C-type lectin receptors.

**Discussion:**

IVIG remodels innate immune communication and activates differential autophagy programs, which may contribute to its therapeutic effects in KD and other autoimmune and inflammatory pathologies.

## 1 Introduction

Autophagy is a versatile cellular degradation machinery that is primarily involved in maintaining cellular homeostasis by removing dysfunctional cellular organelles, aggregates, and misfolded proteins. An increasing amount of evidence collected over the last few decades has expanded the conventional view of autophagy from being a quality control and survival mechanism to a regulator of multifaceted immune processes, including the regulation of immune system functions, inflammatory responses and pathogen elimination (1, 2).

Autophagy has been classified into various types based on the pathways implicated or cargo selectivity. Based on the pathways, autophagy is divided into canonical or classical autophagy pathway (macroautophagy, microautophagy and chaperone-mediated autophagy) and non-canonical pathways (LC3-Associated Phagocytosis (LAP) and LC3-Associated Endocytosis (LANDO)). Macroautophagy is the highly investigated form of autophagy and it begins with activation of ULK1 complex (ULK1/2, ATG13, FIP200, ATG101) upon inhibition of MTORC1 or activation of AMPK pathway. It triggers the nucleation of phagophore membrane by a process dependent on Class III PI3K complex I (VPS34, VPS15, Beclin 1, ATG14L), generating phosphatidylinositol-3-phosphate (PI3P) to recruit downstream effectors. Phagophore expansion requires two critical ubiquitin-like conjugation systems: the ATG12-ATG5-ATG16L1 complex, which functions as an E3 ligase, and the conjugation of phosphatidylethanolamine (PE) to LC3 (producing membrane-anchored LC3-II). The enclosed double-membrane autophagosome ultimately fuses with a lysosome, forming an autolysosome where cargo is degraded by hydrolases. In contrast, non-canonical autophagy pathways involve LC3 lipidated single membrane compartments like endosomes and phagosomes. It operates independently of ULK1 initiation and critically depends on WD40 domain of ATG16L1 and Rubicon associated PI3CK3 complex (3). They are targeted, and regulated in response to extracellular signals.

Autophagy can also be divided into two categories based on cargo selectivity: selective autophagy and nonselective autophagy. Selective autophagy targets specific cellular components like mitochondria (mitophagy), the endoplasmic reticulum (ER-phagy), or protein aggregates (aggrephagy) via adaptor/receptor proteins (e.g., p62, NBR1, OPTN, FUNDC1, NCOA4) that bind cargo and interact with LC3/Atg8 through LIR motifs (4). Nonselective autophagy refers to the bulk transport of organelles or other cytoplasmic components to lysosomes.

Genome-wide association studies (GWAS) have unravelled crucial genetic links between autophagic machinery and various autoimmune and inflammatory diseases (5). Indeed, the unexpected discovery of a Thr300Ala single nucleotide polymorphism in the autophagy-related gene ATG16L1 as a strong genetic predisposition for Crohn’s disease (CD) (6) fuelled a great interest in elucidating the role of defective autophagy in the pathogenesis of these diseases (7). Concurrent with these discoveries, pharmacological targeting of autophagy has been proposed as a potential therapeutic strategy in many diseases, including systemic lupus erythematosus (SLE), inflammatory bowel diseases, rheumatoid arthritis, and multiple sclerosis. A number of direct or indirect autophagy modulators are currently being investigated in clinical trials for the treatment of these conditions, and promising data have been generated (7–9). Furthermore, the therapeutic effects of some of the conventional anti-inflammatory medications such as aspirin and corticosteroids, have now been interpreted at least partly via modulation of the autophagy pathway (10, 11).

One of the mainstay treatments for autoimmune and inflammatory disorders is intravenous immunoglobulin (IVIG), which consists of normal human immunoglobulin G (IgG) obtained from a pool of plasma from several thousand healthy individuals. Although initially used as a replacement therapy in primary immunodeficient patients, a breakthrough observation during early 1980s in paediatric immune thrombocytopenic purpura patients, led to the therapeutic use of IVIG in the large number of autoimmune and inflammatory diseases including immune thrombocytopenic purpura in adults, Kawasaki disease (KD), Guillain–Barré syndrome, chronic inflammatory demyelinating polyneuropathy, and dermatomyositis (12–14). The therapeutic benefits of IVIG could be explained by several mutually non-exclusive mechanisms, including complement scavenging, neutralisation of pathogenic antibodies, blockade of Fc gamma receptors, and regulatory effects on multiple cells of the immune system via distinct pathways (15–25).

Our previous work demonstrated that IVIG induces macroautophagy in inflammatory innate cells like monocytes, M1 macrophages, and dendritic cells (DCs). Induction of macroautophagy was also confirmed in peripheral blood mononuclear cells (PBMC) from IVIG-treated myopathy patients (26). However, it is not known whether IVIG induces only macroautophagy in innate immune cells or if other pathways are also operative. Therefore, by using single cell RNA sequencing data from KD patients treated with IVIG, we investigated the induction of various autophagy pathways across diverse innate immune cell subsets. Our data indicate that while IVIG induces macroautophagy across different innate immune cell subsets, it also activates non-canonical LAP and various selective autophagy pathways in specific innate immune cell subsets of patients with KD. Further mechanistic studies using PBMC from the donors revealed that IVIG-induced autophagy is not restricted to a particular formulation. However, Fc fragment and C-type lectin receptors were found to be dispensable for this process. These results extend our previous findings and provide novel evidence on the differential regulation of autophagy pathways in an innate immune cell-specific manner.

## 2 Material and methods

### 2.1 Analysis of single cell data of Kawasaki patients treated with IVIG

We obtained the single-cell RNA sequencing (ScRNA-seq) data from the GEO public database (https://www.ncbi.nlm.nih.gov/geo/) with accession number GSE168732 (27), which includes PBMC from six KD patients sampled at two time points (acute pre-treatment and 24 h post-IVIG treatment), together with three healthy donors. Thus, the dataset comprises a total of 15 samples, and each sample was processed as an individual Seurat object. The extracted data were in the form of a gene-barcode matrix of (unique molecular identifier) UMI counts. Seurat (v5.0.1) was used (28, 29) for quality control, normalisation, dimensional reduction, batch effect removal, clustering, and visualization. Each sample was converted into a Seurat object, and cells from each sample having counts between 2000 and 60000 having less than 5% mitochondrial genes were selected for further downstream analysis. After filtering low-quality cells, they were log-normalised using the NormalizeData function of Seurat, and the top 2000 variable features were selected for dimensional reduction. All the 15 Seurat objects were integrated using the IntegrateData function in the Seurat package to eliminate any batch effects present in the samples. Canonical correlation analysis (CCA) was used to identify the anchors, and these anchors were used to correct the technical difference between the samples. Later, this integrated Seurat object containing 86074 cells from healthy controls, acute untreated KD patients, and IVIG-treated patients was used for further analysis. After scaling the integrated matrix, the uniform manifold approximation and projection (UMAP) was performed using the top 30 dimensions that emerged from the principal component analysis (PCA). Meanwhile, cell clusters were found using the nearest neighbour graph-based clustering technique on the PCA-reduced data. To obtain the specific innate immune cell subset within PBMC, the resolution was adjusted to 1 and expression of canonical marker genes was then used to annotate cell clusters. The SingleR (v2.8.0) (30) was also used to annotate rare cell populations with the help of the Monnocle immune database. Standard single-cell QC plots (distribution of UMIs, mitochondrial gene percentage, clustering UMAP/t-SNE) and dot plots showcasing marker genes used for annotating innate immune cell subsets were presented in **Supplementary Figures S1–S5**.

A list of core autophagy genes and selective autophagy was curated based on previously published literature (31). To compare the average expression levels of core autophagy and selective autophagy genes across different subsets (e.g., cell types or clusters) within the control, diseased, and IVIG-treated conditions, the average expression data were visualised using the pheatmap (v1.0.12) to generate heatmaps (32). These heatmaps provided a visual comparison of the expression levels of core autophagy genes across different conditions and subsets. The pheatmap function was configured with clustering to highlight similarity in the patterns of gene expression. The expression levels of core autophagy genes were further visualised using box plots to depict the distribution of gene expression across different conditions and immune cell subsets. Significant differences, as determined by the two-sided t-tests, were annotated on the plots.

### 2.2 Differential gene expression analysis

For each cell type, differential expression between treatment groups was assessed using the MAST (Model-based Analysis of Single-cell Transcriptomics) algorithm (v1.32.0) implemented in Seurat. This method accounts for the bimodal distribution and sparsity typical of single-cell data by incorporating cellular detection rate as a covariate in the linear hurdle model. Genes expressed in fewer than 10% of cells were excluded to reduce noise. Differentially expressed genes (DEGs) were defined using thresholds of |log2 fold change| ≥ 0.25 and adjusted p value < 0.05 after Benjamini–Hochberg correction. The resulting DEGs were used for examining the expression of autophagy-related genes across different treatment conditions in innate immune cell subsets.

### 2.3 Correlation analysis of innate immune cell subsets

To evaluate coordinated immune remodeling, Spearman correlation analysis was performed on per-sample cell-type proportions. Correlations were calculated separately for healthy control, untreated KD, and IVIG-treated groups. Correlation matrices were visualized as hierarchical clustering heatmaps using the pheatmap R package. Positive and negative correlations were interpreted as coordinated expansion or reciprocal changes between innate immune cell subsets.

### 2.4 Cell–cell communication analysis

Cell–cell communication analysis was performed using the CellChat R package (v2.2.0) (33) on the integrated single-cell RNA-sequencing dataset of innate immune cells from healthy controls, untreated KD patients, and IVIG-treated patients. Cells were annotated into major innate immune populations, including classical, intermediate, and non-classical monocytes, natural killer (NK) cells, low-density neutrophils, and Vδ2 γδ T cells. For each condition, CellChat objects were constructed independently using the default human ligand–receptor interaction database. Communication probabilities were inferred based on the expression of known ligand–receptor pairs, and statistically significant interactions were identified using permutation testing. Interaction strength was summarized at both the cell–cell level and the pathway level.

To compare global communication patterns across conditions, we visualized (i) heatmaps representing the number of interactions between sender and receiver cell types, (ii) network diagrams illustrating the directionality and strength of intercellular signalling, and (iii) bubble plots highlighting condition-specific ligand–receptor interactions enriched in KD. Plots were generated using standard CellChat visualization functions and CCPlotR R package v(1.4.0) (34).

### 2.5 Functional enrichment analysis of autophagy-related pathways

Gene Ontology (GO) and Kyoto Encyclopedia of Genes and Genomes (KEGG) pathway enrichment analyses were conducted using differentially expressed genes for each innate immune cell subset using clusterProfiler R package (v4.16.0) (35). Enrichment analyses focused on autophagy-, mitophagy-, and vesicular trafficking–related pathways. Dot plots were used to visualize enriched pathways, with dot size representing gene ratios and color indicating adjusted p values.

### 2.6 Pseudotime trajectory analysis of monocyte subsets

Pseudotime analysis was performed exclusively on monocyte subsets using monocle3 R package (v1.4.26) (36) (classical, intermediate, and non-classical monocytes) to infer continuous cell-state transitions. A trajectory inference method compatible with Seurat objects was applied to order cells along a pseudotime axis. Pseudotime distributions were compared across conditions to assess disease- and treatment-associated shifts in monocyte state trajectories.

### 2.7 Reagents and antibodies

Rabbit MAb to β-actin (Clone 13E5, HRP-conjugated), affinity purified antibody to LC3B (#2775), and HRP-linked anti-rabbit IgG (#7074) from Cell Signaling Technology (Ozyme, Saint Quentin Yvelines, France) were used for immunoblotting. Plasma-derived human serum albumin (HSA) was from Laboratoire Française de Biotechnologies (Les Ulis, France).

### 2.8 Preparation of therapeutic IVIG

The IVIG products used for the mechanistic studies were Sandoglobulin®, Gamunex®, Privigen® and Octagam®. IVIG preparations were dialyzed against large volumes of phosphate-buffered saline (PBS) followed by RPMI-1640 at 4 °C for 18 hrs before use. Fc fragments of IVIG were obtained from Dr M. C. Bonnet (Institut Mérieux, Lyon, France) (37). The purity of the Fc fragment was verified by sodium dodecyl sulfate-polyacrylamide gel electrophoresis (SDS-PAGE).

### 2.9 Isolation, culturing and treatment of immune cells from healthy donors

PBMC from healthy donors were isolated by Ficoll density gradient centrifugation of buffy bags purchased from Centre Necker-Cabanel, Etablissement Français du Sang, Paris, France (Institut National de la Santé et de la Recherche-EFS ethical committee convention 15/EFS/012; 18/EFS/033) (26). Human CD14 MicroBeads (Miltenyi) were used to isolate monocytes from the PBMC.

PBMC and monocytes (0.5 million cells/ml) were cultured in RPMI-1640 supplemented with 10% fetal calf serum, and 1% penicillin-streptomycin. PBMC were treated *in vitro* with different IVIG products (25 mg/ml) or equimolar concentrations (0.15 mM) of Fc fragment of IVIG or HSA (as an irrelevant protein control) until the indicated time points. To explore the role of C-type lectin receptors (CLRs), monocytes were treated with the calcium chelating agent ethylenediamine tetra acetic acid (EDTA, 0.5 mM) for one hour before culture with IVIG (25 mg/ml) for 24 hrs.

### 2.10 Immunoblotting analysis

PBMC or monocytes from healthy donors were treated *in vitro*, pelleted, and lysed in RIPA buffer (Sigma) supplemented with protease and phosphatase inhibitors (Roche Biotech) on ice for 30 min. Protein concentrations in whole-cell lysates were quantified, and equal amounts from each sample were resolved by SDS-PAGE, followed by transfer onto polyvinylidene fluoride (PVDF) membranes using a wet western blotting method. Membranes were blocked, incubated with primary antibodies and incubated with HRP-conjugated anti-rabbit IgG secondary antibody as described previously (26). SuperSignal™ West Dura Extended Duration substrate (Thermo Fisher Scientific, Courtaboeuf, France) was used for developing the blots. β-actin served as a loading control. myImageAnalysis software v2.0 (Thermo Fisher Scientific) was used for the analyses of Western blot images. Full western blot images are presented in **Supplementary Figure S6**.

### 2.11 Statistics

As described in the figure legends, the studies were conducted using PBMC or monocytes from multiple independent donors. Data on the expression of autophagy genes were compared using two-sided Wilcoxon rank sum tests for pairwise comparison in R using ggpubr R package (0.6.0). Immunoblot data were analysed by one-way analysis of variance (ANOVA) with Dunnett’s multiple comparison test by using Prism 8 (GraphPad Software Inc., CA).

## 3 Results

### 3.1 Remodelling of the innate immune cell landscape following IVIG therapy in Kawasaki disease

**Figure 1:**
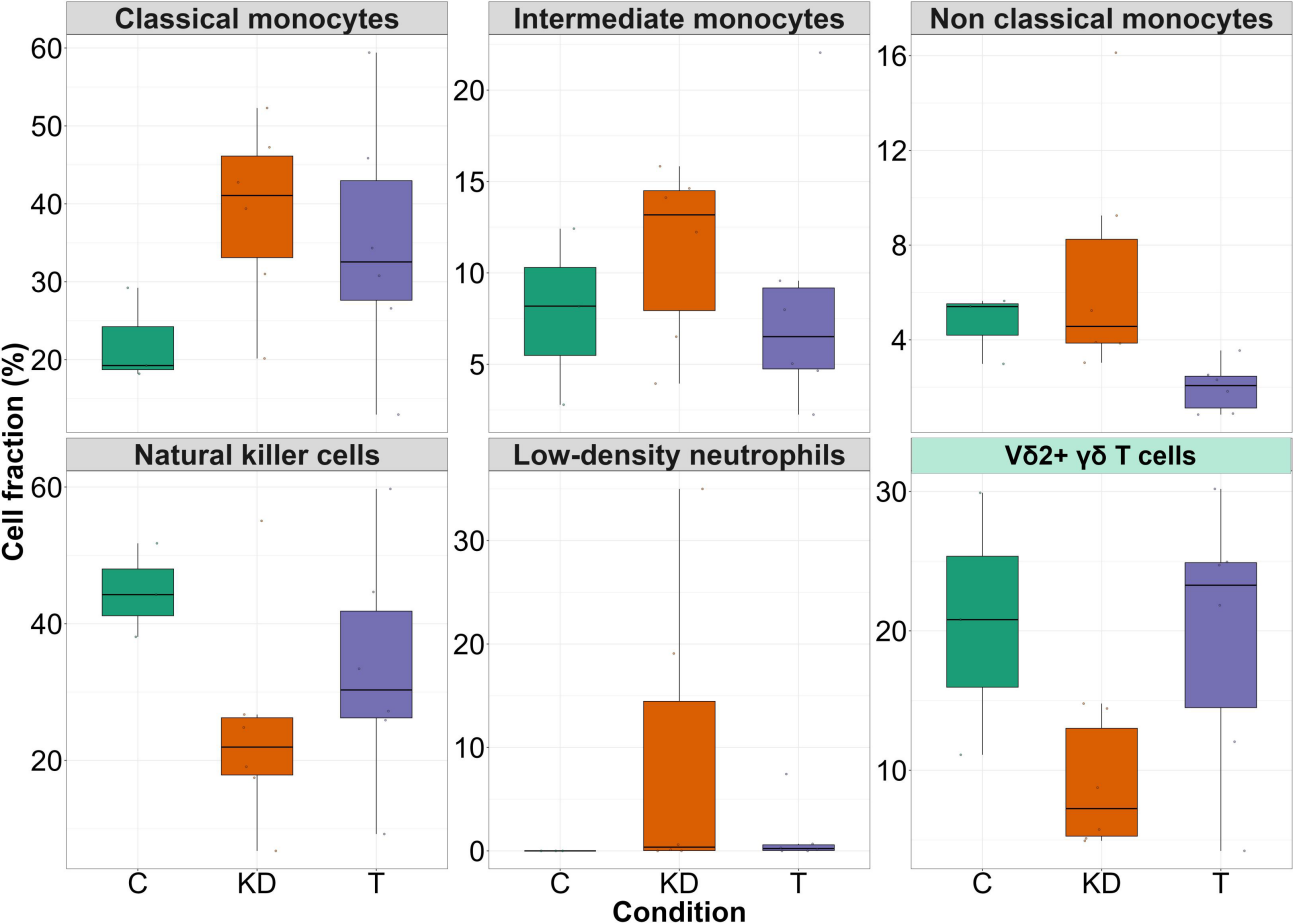
The frequencies of innate immune cell subsets in patients with Kawasaki disease and following IVIG treatment. Boxplots representing the proportion (%) of innate immune cell subsets—including classical, intermediate, and non-classical monocytes, low-density neutrophils, natural killer (NK) cells, and γδ T cells—across three conditions: Control (C) (n = 3 donors), Kawasaki disease (KD) patients (n = 6 patients), and IVIG-treated KD patients (T) (n = 6 patients).

We examined the distribution of innate immune cell subsets across healthy individuals (C), untreated KD patients (KD), and IVIG-treated patients (T) using single-cell transcriptomic data. Distinct alterations in cell proportions were observed between disease and treatment conditions (**Figure 1**).

Classical monocytes were markedly expanded in KD patients compared to healthy controls, but their frequency declined following IVIG treatment, approaching baseline levels. A similar trend was evident for intermediate monocytes, which were elevated in untreated patients and reduced post-IVIG treatment. Non-classical monocytes were also increased in patients but were nearly absent after IVIG therapy, suggesting a selective suppression of this pro-inflammatory subset. Low-density neutrophils, which were undetectable in controls, accumulated prominently in KD and were decreased following IVIG therapy, consistent with their role in systemic inflammation. In contrast, NK cells displayed the opposite pattern: they were reduced in KD patients but partially restored upon treatment, highlighting an immunoregulatory effect of IVIG. Finally, Vδ2+γδ T cells were diminished in disease compared with controls, but their frequency increased following treatment, indicating recovery of this protective lymphocyte subset.

Together, these findings, in line with previous studies (38–40), revealed a pronounced reshaping of the innate immune landscape in KD, characterized by expansion of inflammatory monocytes and neutrophils, and a concomitant loss of NK and γδ T cells. IVIG therapy reverses these alterations.

### 3.2 Correlation analysis between changes in different cell subsets to infer coordinated immune remodeling

**Figure 2:**
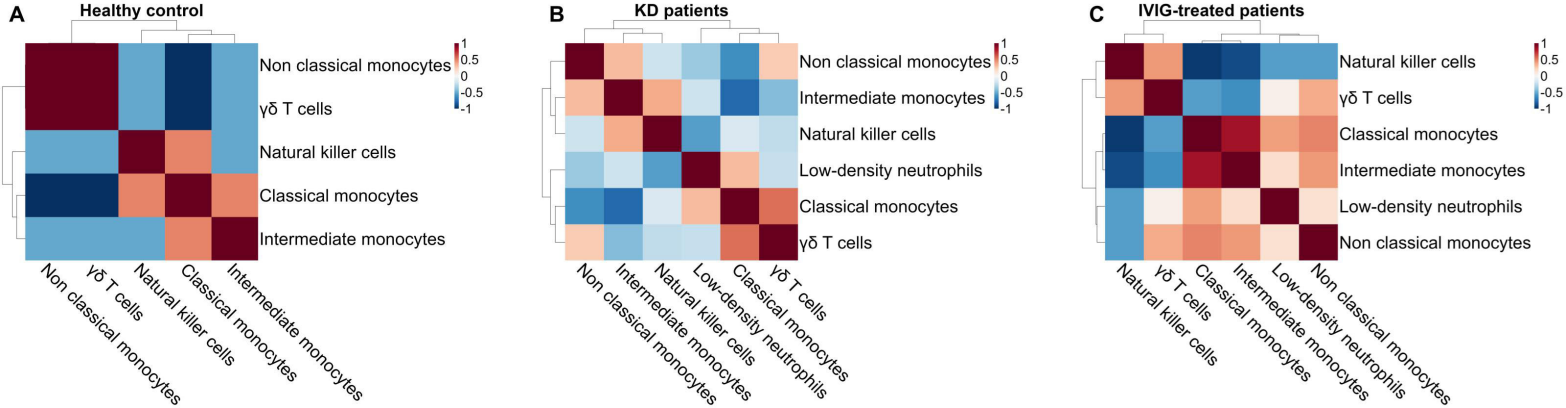
Coordinated innate immune remodeling in Kawasaki disease and its modulation by IVIG. Spearman correlation heatmaps illustrate relationships between per-patient proportions of innate immune cell subsets in **(A)** healthy controls (n = 3 donors), **(B)** untreated acute Kawasaki disease (KD) patients (n = 6 patients), and **(C)** IVIG-treated KD patients (n = 6 patients). Color gradients indicate the strength and direction of correlations (red, positive; blue, negative).

To determine whether changes in innate immune cell frequencies occurred in a coordinated manner, we performed Spearman correlation analysis of per-patient cell-type proportions across conditions. In healthy controls, correlations among innate immune subsets were generally weak, consistent with independent variation of immune compartments under homeostatic conditions (**Figure 2A**).

In contrast, KD patients exhibited pronounced restructuring of correlation patterns, characterized by strong positive correlations among low-density neutrophils, classical monocytes, and intermediate monocytes, alongside strong negative correlations between these myeloid populations and cytotoxic innate lymphocytes, including NK cells and Vδ2 γδ T cells (**Figure 2B**). These findings indicate coordinated expansion of inflammatory myeloid subsets coupled with reciprocal contraction of cytotoxic innate populations in KD.

Notably, IVIG-treated KD patients displayed partial normalization of these relationships, with attenuation of both positive myeloid correlations and negative correlations with NK and Vδ2+γδ T cells (**Figure 2C**), suggesting restoration of immune network balance following treatment.

Together, these analyses demonstrate that KD is associated with a coordinated remodeling of the innate immune landscape, characterized not only by altered cell-type proportions but also by disease-specific restructuring of inter-cellular relationships. The emergence of tightly coupled myeloid expansion and reciprocal suppression of cytotoxic innate lymphocytes underscores a polarized inflammatory network in KD, which is partially reversed following IVIG treatment. These findings provide systems-level evidence that KD involves functional reorganization of innate immunity rather than isolated changes in individual cell subsets.

### 3.3 Global remodeling of innate immune cell communication in KD patients

**Figure 3:**
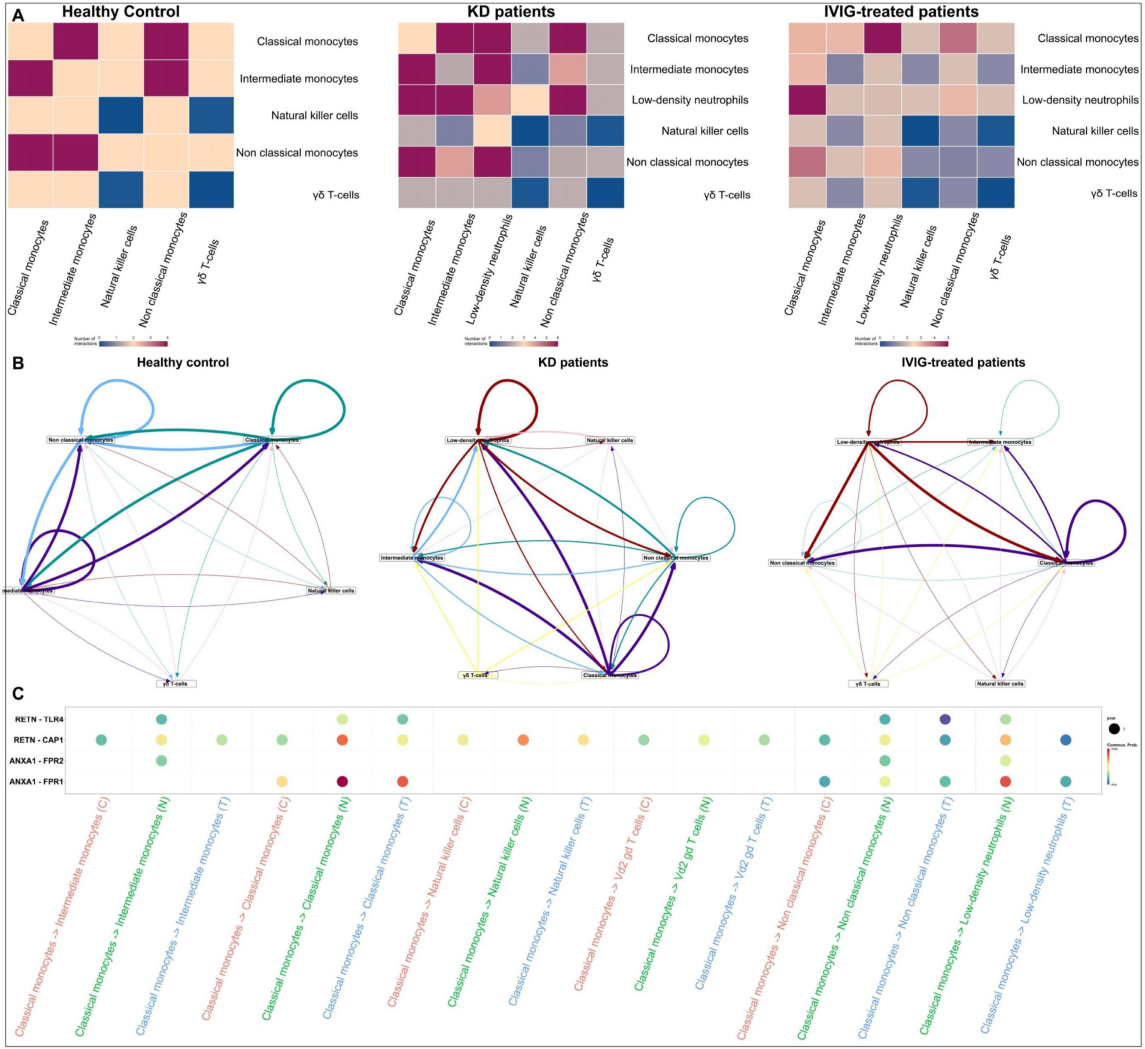
Remodeling of innate immune cell–cell communication in Kawasaki disease and following IVIG treatment **(A)** Heatmaps showing the number of inferred cell–cell interactions among innate immune populations in healthy controls (n = 3 donors), Kawasaki disease (KD) patients (n = 6 patients), and IVIG-treated KD patients (n = 6 patients), as identified by CellChat analysis. Rows represent receiver cell types and columns represent sender cell types. **(B)** Network diagrams illustrating the global architecture, directionality, and strength of intercellular communication in each condition. Nodes represent innate immune cell types, while edges indicate ligand–receptor–mediated signaling interactions. Edge thickness corresponds to interaction strength. **(C)** Bubble plot depicting representative ligand–receptor interactions significantly enriched in KD. The x-axis indicates sender–receiver cell pairs, and the y-axis shows selected ligand–receptor pairs. Bubble size reflects the communication probability, while color intensity denotes statistical significance.

Heatmap visualization revealed substantial differences in cell–cell communication patterns across healthy, acute untreated KD, and IVIG-treated patients (**Figure 3A**). In healthy controls, interactions among innate immune subsets were relatively balanced, with moderate communication involving monocyte subsets and NK cells. In contrast, KD patients showed a marked increase in interaction density, particularly involving classical and intermediate monocytes, low-density neutrophils, and NK cells, indicating heightened inflammatory crosstalk during disease. Several monocyte-centered interactions were selectively amplified in KD, consistent with aberrant activation of the innate immune compartment. Network visualization further highlighted condition-specific differences in communication architecture (**Figure 3B**). In healthy controls, signaling networks were relatively sparse and evenly distributed across cell types. In KD, however, monocyte subsets emerged as dominant signaling hubs, with strong outgoing and incoming interactions connecting monocytes to neutrophils, NK cells, and γδ T cells.

Further, bubble plot analysis identified specific ligand–receptor interactions that were significantly enriched in KD patients compared with healthy and IVIG-treated patients (**Figure 3C**). These interactions predominantly involved monocyte-derived inflammatory signaling, including pathways associated with resistin, annexin, and TLR-related signaling, which are known upstream regulators of pro-inflammatory cytokines such as TNF, IL-1β, and IL-6.

The strength and frequency of these KD-specific interactions were reduced or redistributed following IVIG treatment, supporting a role for IVIG in dampening excessive innate immune activation and restoring communication toward a homeostatic state.

### 3.4 IVIG treatment leads to induction of macroautophagy and noncanonical LAP pathways in innate immune cells of KD patients

Our previous work has illustrated the induction of canonical macroautophagy by IVIG in peripheral blood inflammatory innate immune cells such as monocytes, dendritic cells, and M1 macrophages, and also in treated inflammatory myopathy patients (26). Therefore, by using single-cell transcriptomic data, we first investigated the induction of autophagy genes in innate immune cell subsets of IVIG-treated KD patients.

**Figure 4:**
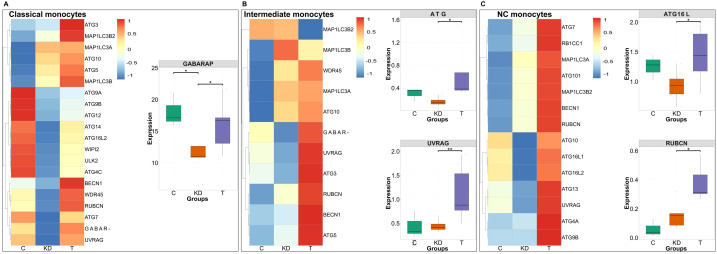
Expression of autophagy-related genes across monocyte subsets in patients with Kawasaki disease and following IVIG treatment. Heatmaps **(A–C)** displaying the standardized expression (z-score) of autophagy-related genes in classical **(A)**, intermediate **(B)**, and non-classical (NC) monocytes **(C)** across three conditions: healthy controls (C; n = 3 donors), Kawasaki disease (KD; n = 6 patients), and IVIG-treated KD patients (T; n = 6 patients). Warmer colors (red) represent higher expression levels, while cooler colors (blue) indicate lower expression. The genes that show significant changes in the expression following IVIG therapy are presented separately in the form of boxplots. Each dot represents an individual patient sample. Boxes indicate the interquartile range (IQR), lines show medians, and whiskers represent 1.5× IQR. Two-sided Wilcoxon rank sum tests were used to calculate significance*P < 0.05; **P < 0.01.

Among the monocyte subsets, classical monocytes exhibited increased expression of GABARAP, BECN1, ATG2B, and MAP1LC3B2 and other macroautophagy genes following 24 hours of IVIG therapy, with a particularly significant upregulation of GABARAP, consistent with its known induction in macroautophagy-activated cells (31) (**Figure 4A**). Intermediate monocytes exhibited elevated expression of majority of the autophagy-related genes with significant upregulation of ATG7 and UVRAG, which are implicated in the extension of autophagosome and its fusion with lysosome during macroautophagy (41) (**Figure 4B**). Of note, in non-classical monocytes, IVIG significantly enhanced the expression of RUBCN and ATG16L2 (**Figure 4C**). RUBCN is known to be negative regulator of canonical autophagy and plays a critical role in LC3 induced phagocytosis (LAP), which relies on components of the autophagy machinery particularly, ATG16L2 for processing the extracellular cargo (42).

**Figure 5:**
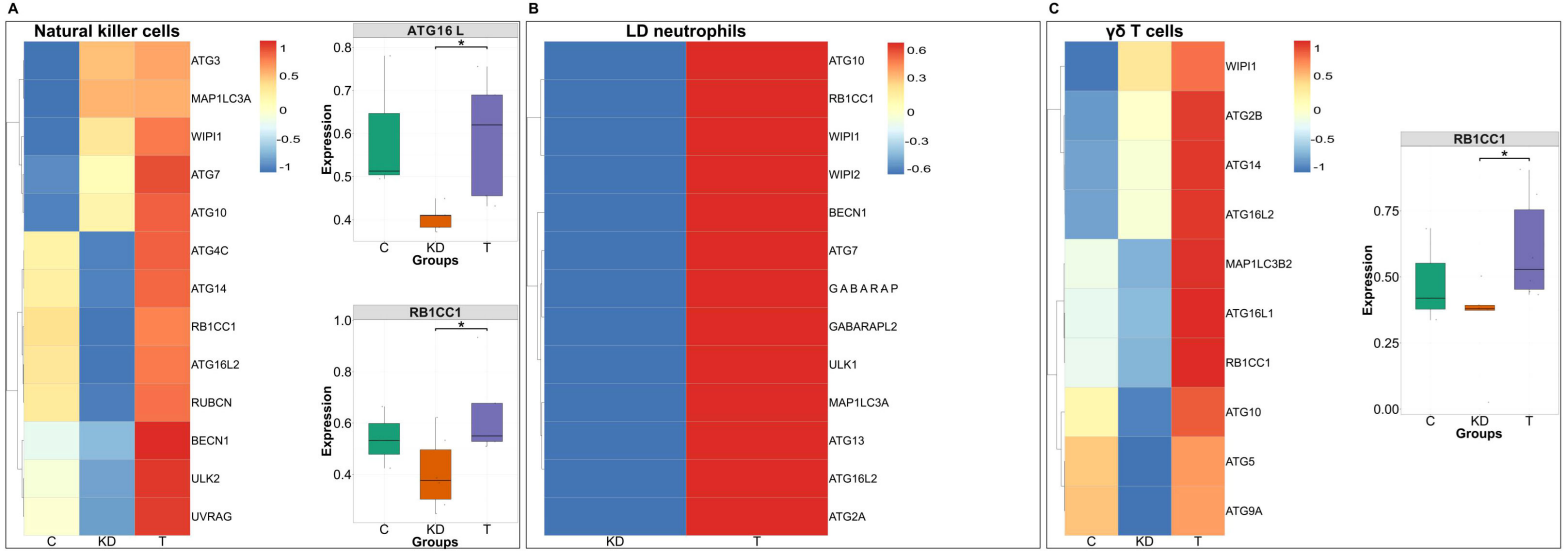
Expression of autophagy-related genes across NK cells, low density neutrophils and γδ T cells in patients with Kawasaki disease following IVIG therapy. Heatmaps **(A–C)** displaying the standardized expression (z-score) of autophagy-related genes in NK cells **(A)**, Low density (LD) neutrophils **(B)**, and γδ T cells **(C)** across three conditions: healthy controls (C; n = 3 donors), Kawasaki disease (KD; n = 6 patients), and IVIG-treated KD patients (T; n = 6 patients). Warmer colors (red) represent higher expression levels, while cooler colors (blue) indicate lower expression. The genes that show significant changes in the expression following IVIG therapy are presented separately in the form of boxplots. Each dot represents an individual patient sample. Boxes indicate the interquartile range (IQR), lines show medians, and whiskers represent 1.5× IQR. Two-sided Wilcoxon rank sum tests were used to calculate significance *P < 0.05.

NK cells also exhibited upregulation of core macroautophagy genes following IVIG therapy, with a significant increase in RB1CC1 and ATG16L2 (**Figure 5A**). Low density neutrophils, which are activated in inflammatory conditions such as KD and contribute to inflammation, showed increased autophagy gene expression after IVIG therapy (**Figure 5B**). Similarly, γδ T cells displayed induction of autophagy with elevated expression of autophagy genes and significant upregulation of RB1CC1 (**Figure 5C**).

Overall, these results demonstrate that IVIG induces macroautophagy across diverse innate immune cell subsets in KD patients, thereby validating our previous findings and extending them to an inflammatory disease setting.

### 3.5 Differential induction of selective autophagy in innate immune cells following IVIG therapy in KD patients

**Figure 6:**
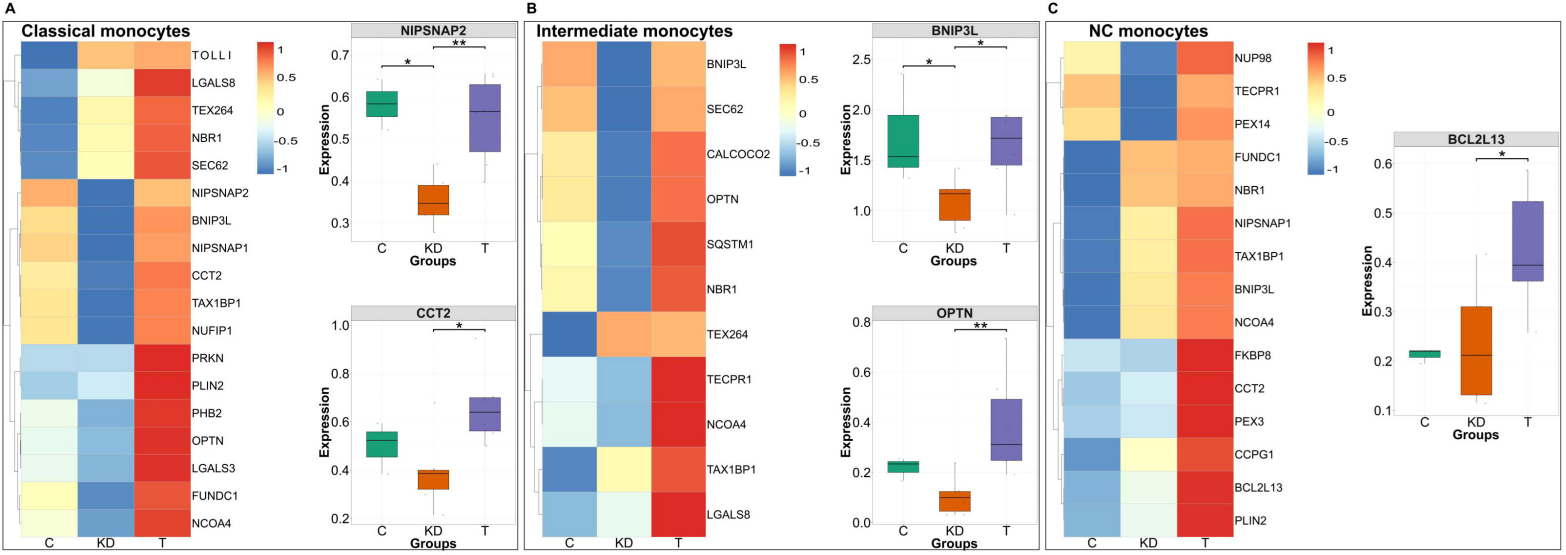
Differential autophagy cargo receptor expression in monocyte subsets of Kawasaki disease and in IVIG-treated patients. Heatmaps **(A–C)** displaying the standardized expression (z-score) of autophagy-related genes in classical **(A)**, intermediate **(B)**, and non-classical (NC) monocytes **(C)** across three conditions: healthy controls (C; n = 3 donors), Kawasaki disease (KD; n = 6 patients), and IVIG-treated KD patients (T; n = 6 patients). Warmer colors (red) represent higher expression levels, while cooler colors (blue) indicate lower expression. The genes that show significant changes in the expression following IVIG therapy are presented separately in the form of boxplots. Each dot represents an individual patient sample. Boxes indicate the interquartile range (IQR), lines show medians, and whiskers represent 1.5× IQR. Two-sided Wilcoxon rank sum tests were used to calculate significance *P < 0.05; **P < 0.01.

**Figure 7:**
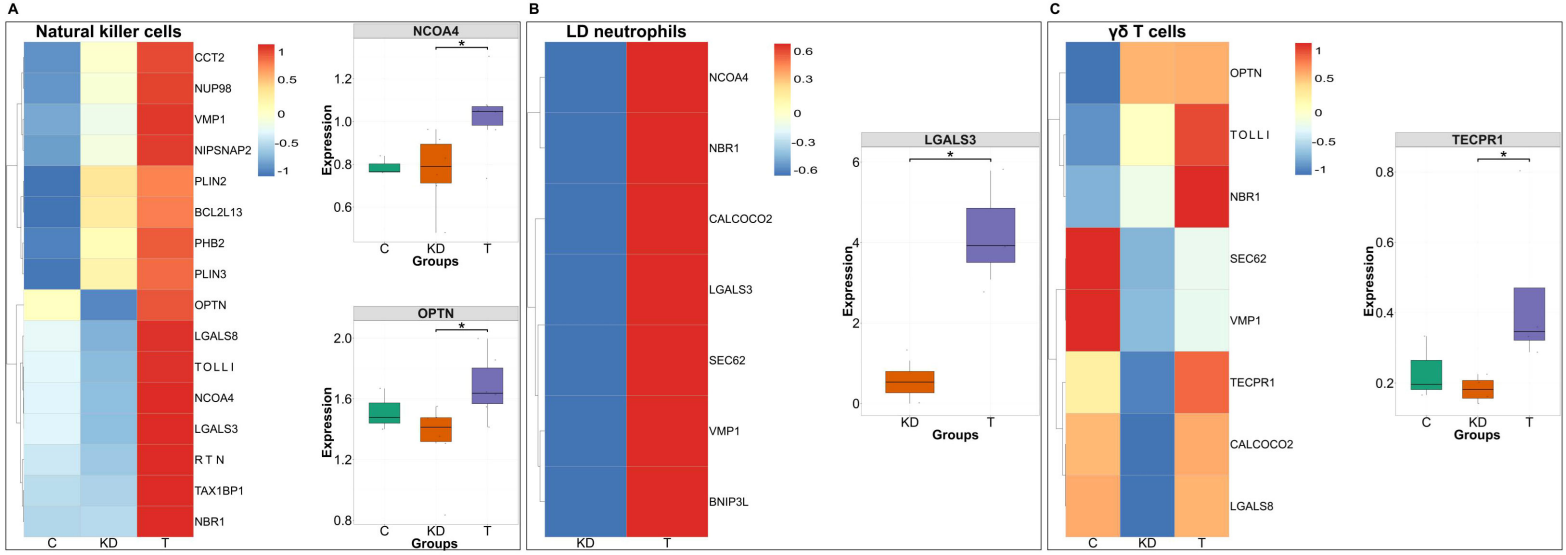
Differential autophagy cargo receptor expression across NK cells, low density neutrophils and γδ T cells subsets in Kawasaki disease and following IVIG treatment. Heatmaps **(A–C)** displaying the standardized expression (z-score) of autophagy-related genes in NK cells **(A)**, low density (LD) neutrophils **(B)**, and γδ T cells **(C)** across three groups: healthy controls (C; n = 3 donors), Kawasaki disease (KD; n = 6 patients), and IVIG-treated patients (T; n = 6 patients). Warmer colors (red) represent higher expression levels, while cooler colors (blue) indicate lower expression. The genes that show significant changes in the expression following IVIG therapy are presented separately in the form of boxplots. Boxes indicate the interquartile range (IQR), lines show medians, and whiskers represent 1.5× IQR. Two-sided Wilcoxon rank sum tests were used to calculate significance *P < 0.05.

To investigate if IVIG therapy induces distinct forms of selective autophagy in different innate immune cell subsets, we analyzed the gene expression of key selective autophagy cargo receptors and adaptor proteins (**Figures 6**, **7**). This approach enabled us to delineate the specific selective autophagy pathways triggered in different innate immune cells following IVIG therapy.

In classical monocytes, we observed significantly increased expression of NIPSNAP2 and CCT2, which mediate the selective degradation of damaged or depolarized mitochondria and protein aggregates, respectively (43, 44) (**Figure 6A**). In intermediate monocytes, IVIG therapy led to significant upregulation of mitophagy-related genes such as OPTN and BNIP3L, which participate in parkin-induced mitophagy by binding to ubiquitin chains on damaged mitochondrial membranes and recruiting autophagy proteins (**Figure 6B**). Non-classical monocytes showed significant induction of BCL2L13, a mitophagy cargo receptor that promotes DNM1L-mediated mitochondrial fission (45) (**Figure 6C**).

In NK cells, IVIG therapy significantly increased the expression of OPTN, a mitophagy adaptor, and NCOA4, a ferritin cargo receptor that promotes ferritinophagy and the release of free iron into the cytosol (**Figure 7A**). Low density neutrophils displayed broad upregulation of cargo receptors, including a marked increase in LGALS3, a xenophagy receptor that targets cytosolic pathogens for autophagic degradation (46) (**Figure 7B**). γδ T cells exhibited enhanced expression of multiple cargo proteins, with a notable increase in TECPR1, which facilitates the clearance of aggregated proteins (47, 48) (**Figure 7C**).

Thus, in addition to inducing macroautophagy and LC3-associated phagocytosis (LAP), IVIG therapy also triggers diverse selective autophagy pathways across innate immune cell subsets.

### 3.6 Cell-type-specific enrichment of autophagy-related pathways across innate immune subsets

**Figure 8:**
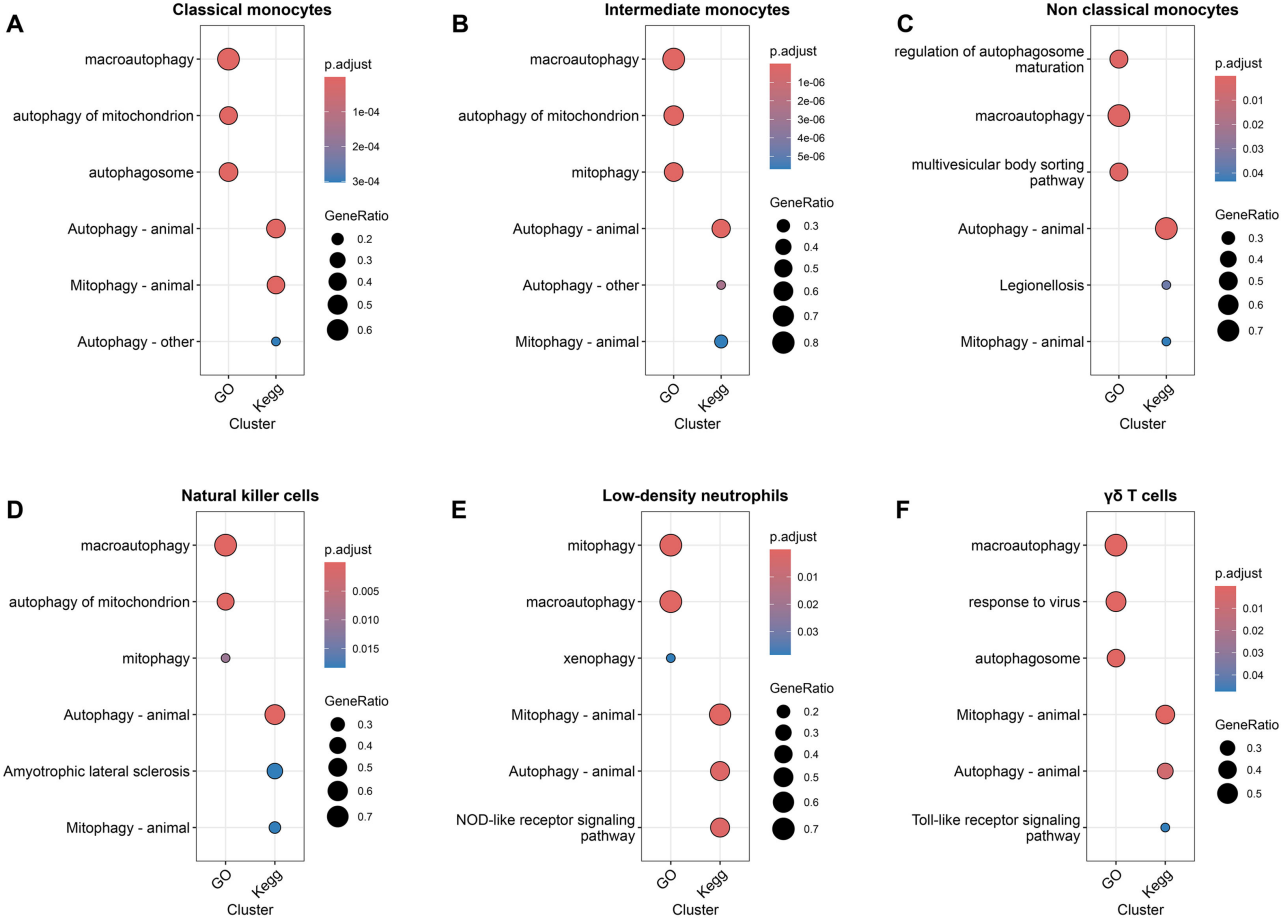
Cell-type-specific enrichment of autophagy-related pathways across innate immune populations in IVIG-treated KD patients compared to acute untreated KD patients. Dot plots showing GO and KEGG pathway enrichment of autophagy-related gene sets in **(A)** classical monocytes, **(B)** intermediate monocytes, **(C)** non-classical monocytes, **(D)** natural killer cells, **(E)** low-density neutrophils, and **(F)** γδ T cells. Dot size represents the GeneRatio, and color indicates adjusted p values.

To investigate functional autophagy reprogramming of innate immune populations in IVIG-treated KD patients compared to acute untreated KD patients, we performed Gene Ontology (GO) and KEGG pathway enrichment analyses using differentially expressed genes from each cell subset (**Figure 8**). Pathway enrichment analysis revealed widespread but cell-type-specific activation of autophagy-related programs across innate immune populations. Classical and intermediate monocytes displayed strong enrichment of macroautophagy, mitophagy, and autophagosome-associated pathways (**Figures 8A, B**), consistent with heightened metabolic and inflammatory activity. Non-classical monocytes showed enrichment of pathways related to regulation of autophagosome maturation and vesicular trafficking, indicating a more regulatory autophagy profile (**Figure 8C**).

In addition to monocytes, low-density neutrophils exhibited pronounced enrichment of mitophagy and xenophagy pathways, together with innate immune signaling pathways (**Figure 8E**), suggesting integration of mitochondrial quality control with inflammatory responses. NK cells and γδ T cells also demonstrated enrichment of autophagy- and mitophagy-related pathways, albeit with lower gene ratios (**Figures 8D, F**), consistent with metabolic adaptation and functional reprogramming of cytotoxic innate lymphocytes. Collectively, these results indicate that autophagy-related processes are broadly engaged across innate immune subsets, with distinct pathway usage reflecting cell-type-specific functional states.

### 3.7 IVIG-induced autophagy coincides with normalization of monocyte pseudotime states

**Figure 9:**
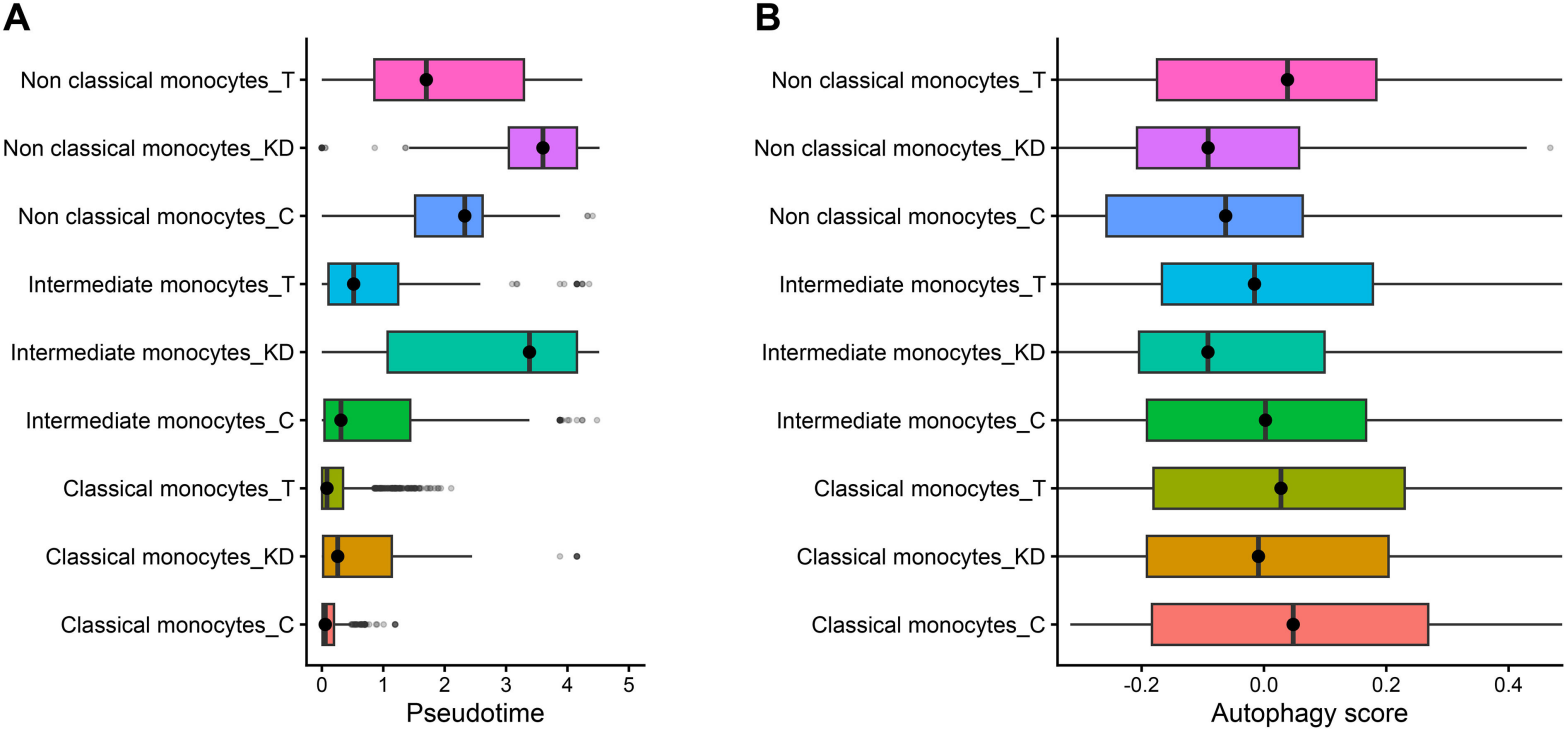
IVIG-induced autophagy concurs with normalization of monocyte pseudotime states **(A)** Distribution of classical, intermediate, and non-classical monocytes from control **(C)** (n = 3 donors), Kawasaki disease (KD) (n = 6 patients), and IVIG-treated (T) KD patients (n = 6 patients), along a pseudotime trajectory inferred from monocyte subsets. **(B)** Autophagy gene signature scores across the aforementioned monocyte subsets, showing increased autophagy activity following IVIG treatment across all subsets.

Pseudotime analysis of monocyte subsets showed that monocytes from KD patients were preferentially distributed toward later pseudotime states, indicating altered monocyte activation compared with controls (**Figure 9A**). Following IVIG treatment, monocyte subsets shifted toward earlier pseudotime values, approaching control-like distributions and suggesting normalization of monocyte state trajectories.

Consistent with this shift, autophagy gene signature scores were increased in monocytes from IVIG-treated KD patients across classical, intermediate, and non-classical subsets (**Figure 9B**). Importantly, increased autophagy in monocytes from IVIG-treated KD patients was associated with the restoration of pseudotime distributions toward homeostatic states. Together, these results indicate that IVIG-induced autophagy is linked to the normalization of monocyte phenotypes in KD.

### 3.8 Kinetics of IVIG-induced autophagy

While these data strongly suggested engagement of the autophagy machinery at the transcriptional level, they did not provide temporal resolution regarding when autophagy is initiated by IVIG or whether its activation is formulation-specific. Defining the timing of autophagy induction is particularly important for identifying optimal windows for downstream mechanistic studies and for distinguishing early autophagy signaling events from later-stage cellular responses such as cargo degradation or cytokine modulation.

**Figure 10:**
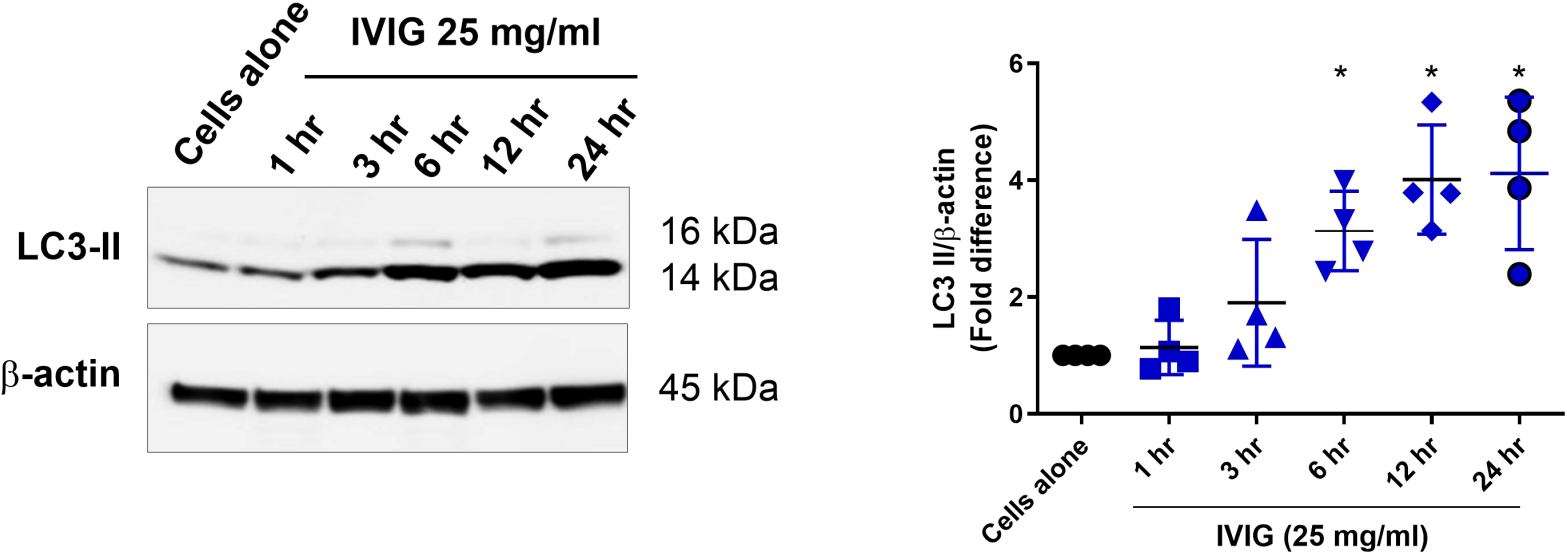
IVIG induces autophagy in PBMC in a time-dependent manner. PBMC from healthy donors were treated *in vitro* with IVIG (Privigen; 25 mg/ml) for different time points (1, 3, 6, 12, and 24hrs). Immunoblot analysis of LC3-II levels is shown. The data (mean ± SD) were from four independent experiments. Statistical significance as determined by one–way ANOVA with Dunnett’s multiple comparisons post-test. *P < 0.05.

To address this, we investigated the kinetics of IVIG-induced autophagy *in vitro* in PBMC isolated from healthy individuals by analyzing LC3-II protein levels. Cells were treated with IVIG (Privigen; 25 mg/ml) for different time points (1, 3, 6, 12, and 24 hours), followed by treatment with bafilomycin (50 nM) for 45 minutes. We observed that IVIG significantly increased LC3-II levels by 6 hours, which peaked at 12 hours and remained elevated at 24 hours (**Figure 10**).

### 3.9 Effect of different IVIG formulations on induction of autophagy

**Figure 11:**
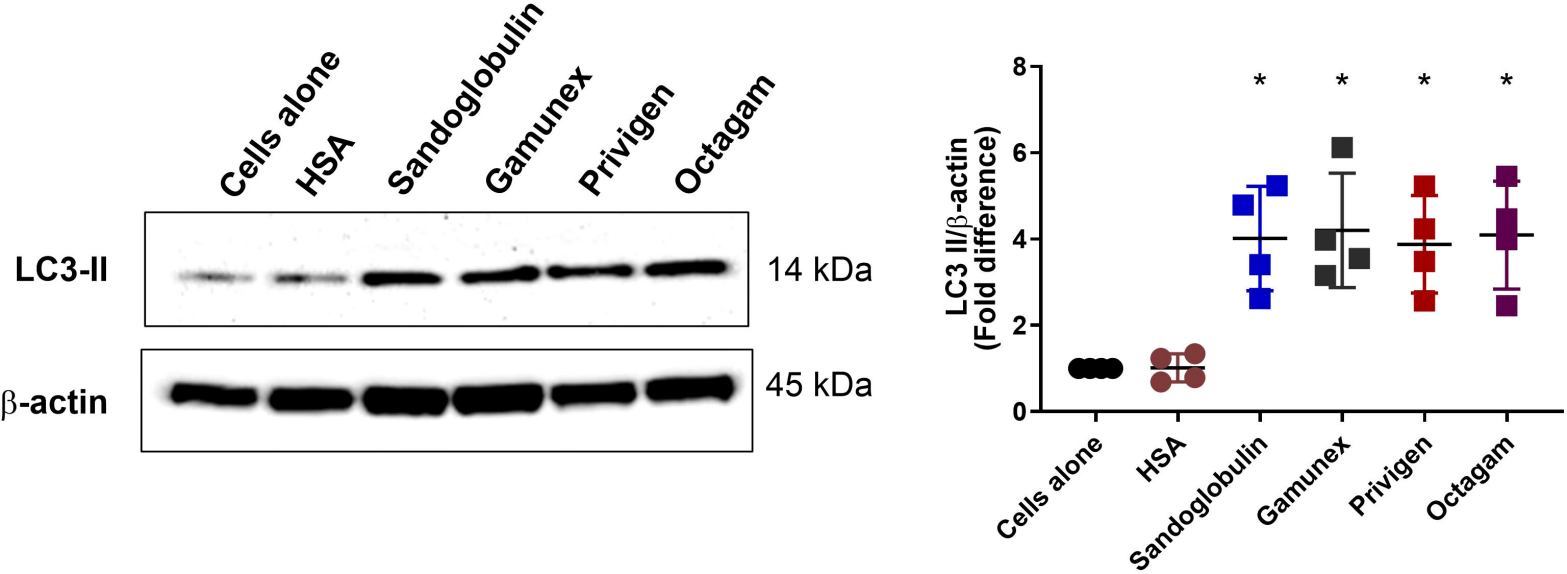
Various IVIG formulations induce autophagy in PBMC in a similar manner. PBMC from healthy donors were exposed to various IVIG formulations ie., Sandoglobulin®, Gamunex®, Privigen®, and Octagam® (25 mg/ml) *in vitro* for 24 hours. Equimolar concentrations of HSA was used as an irrelevant protein control. Immunoblot analysis of LC3-II levels is shown. The data (mean ± SD) were from four independent experiments. Statistical significance as determined by one–way ANOVA with Dunnett’s multiple comparisons post-test. *P < 0.05.

IVIG products exhibit variability in plasma source, IgG purification methods, stabilizing agents, and formulation (49, 50). We therefore asked whether IVIG induces autophagy irrespective of the therapeutic preparation. To address this, we compared the ability of different IVIG formulations: Sandoglobulin^®^, Gamunex^®^, Privigen^®^, and Octagam^®^ to induce autophagy. Treatment of PBMC *in vitro* with each IVIG preparation (25 mg/ml, 24 hours) resulted in a comparable upregulation of LC3-II proteins, indicating that autophagy induction is not restricted to a specific IVIG formulation but rather represents a universal phenomenon independent of source or manufacturing process (**Figure 11**).

### 3.10 Fc fragment and C-type lectin receptors are dispensable for IVIG-induced autophagy in PBMC

**Figure 12:**
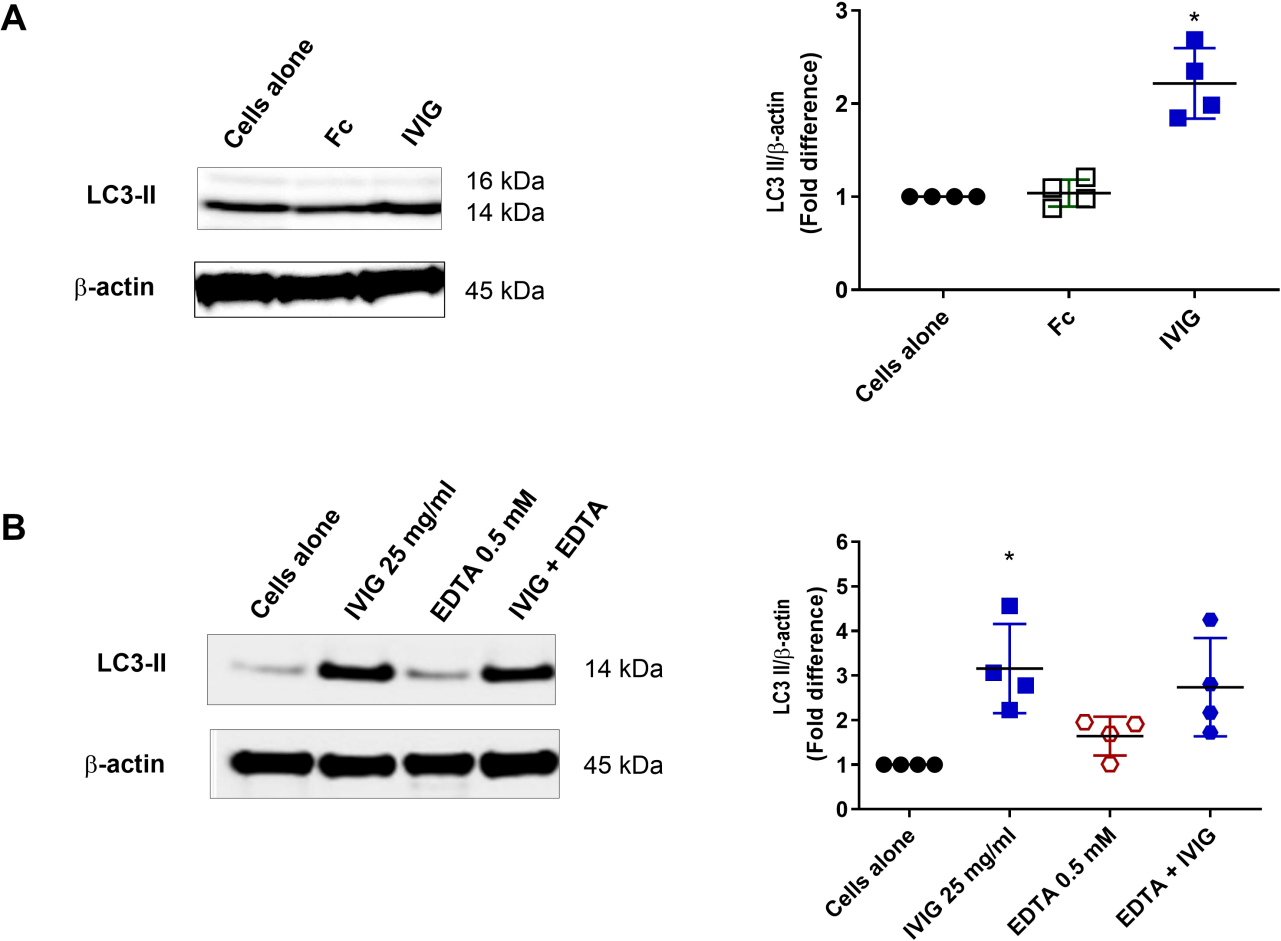
Fc fragment and C-type lectin receptors are dispensable for IVIG-induced autophagy. **(A)** Immunoblotting for LC3-II on lysates of healthy donors’ PBMC treated *in vitro* with IVIG (Privigen; 25 mg/ml) or equimolar concentration (9 mg/ml) of Fc fragment. **(B)** Monocytes were pre-treated with EDTA for 1 hr before culture with IVIG (Privigen; 25 mg/ml) for 24 hrs, followed by immunoblotting for LC3-II. The data (mean ± SD) were from four independent experiments. Statistical significance as determined by one–way ANOVA with Dunnett’s multiple comparisons post-test. *P < 0.05.

IgG consists of an F(ab’)_2_ fragment and an Fc fragment. Some mechanisms of IVIG are mediated through the F(ab’)_2_ region, while others depend on the Fc portion (15–24). Our previous data showed that F(ab’)_2_ fragments of IVIG can induce autophagy in PBMC (26). To determine whether Fc fragments also contribute, we exposed PBMC from healthy donors *in vitro* to either IVIG (Privigen; 25 mg/ml) or equimolar concentrations of Fc fragments (9 mg/ml) for 24 hours. Immunoblot analysis of LC3-II levels revealed that Fc fragments are dispensable for autophagy induction (**Figure 12A**).

Glycosylation of IgG, particularly sialylation of the F(ab’)_2_ or Fc fragments, is critical for several IVIG effector functions, at least partly via glycoprotein-binding cell surface receptors of the calcium-dependent C-type lectin family (51–54). However, sialylation-independent effects of IVIG have also been reported (55–58). Consistent with this, our experiments with desialylated IVIG confirmed that sialylation is not required for autophagy induction (26). This does not, however, exclude the potential involvement of other C-type lectin receptors (CLRs) in a sialylation-independent manner (59). To examine this, we treated monocytes with the calcium chelating agent EDTA (0.5 mM) for one hour before culturing the PBMC with IVIG (Privigen; 25 mg/ml) for 24 hrs. We found that CLR inhibition had no repercussion on IVIG-induced autophagy and LC3-II levels were comparably upregulated in IVIG-treated cells with or without prior treatment of PBMC with EDTA, indicating that CLRs are not implicated in IVIG-induced autophagy in monocytes (**Figure 12B**).

## 4 Discussion

Autophagy plays a pivotal role in the normal functioning of the immune system as a driver of immune homeostasis. It regulates innate inflammatory immune responses by inhibition of inflammasome responses or type-1 interferon signalling against pathogen or damage-associated stimuli by clearance of cytoplasmic ligands (41). Studies in monocytes have demonstrated that suppression of autophagy increases IL-1β production as ATG16L1 deficiency results in increased inflammatory cytokines IL-1β and IL-18. Likewise, downregulation of ATG7 and LC3 leads to increased levels of IL-1β and pyroptosis (41). Dysregulation of autophagy can lead to immune disorders and contribute to autoimmune and inflammatory diseases (60). In fact, KD is often associated with hyperactive innate immune responses, and elevated levels of proinflammatory molecules and oxidative stress-related markers are characteristic features of acute phase of KD (61). IVIG therapy is considered as a standard first-line therapy for KD (61). Our previous study has demonstrated an induction of autophagy by IVIG in inflammatory innate immune cells such as monocytes, M1 macrophages and DCs (26). We further investigated the single cell transcriptomics data of KD patients treated with IVIG to investigate the various autophagic pathways induced by IVIG in different innate immune cell subsets.

The preferential expansion of inflammatory monocyte subsets and low-density neutrophils in KD patients is consistent with heightened innate immune activation and systemic inflammation, processes implicated in vascular injury and coronary artery involvement (62–64). Conversely, the reduction of NK and γδ T cells may reflect impaired cytotoxic and immunoregulatory functions, potentially contributing to inadequate control of inflammation during acute disease (65). Restoration of these cytotoxic innate lymphocyte populations following IVIG treatment further supports an immunomodulatory role for therapy in rebalancing innate immune responses (61).

As evidenced by our analyses and reported elsewhere (27, 38–40), KD patients display a larger proportion of monocytes and low-density neutrophils compared to healthy individuals. Also, downregulation of autophagy is seen in inflammatory diseases like KD (60, 66, 67). PBMC from KD patients show reduced mRNA levels of LC3, BECN1, and ATG16L1 compared to controls (66, 68, 69). γδ T cells, which are hyperactivated in KD, also show reduced autophagy flux with increased IL-17 production (70, 71). These studies thus suggest that reduced autophagy flux in innate immune cells is associated with inflammation and immune dysregulation in inflammatory conditions. Our findings clearly show elevated expression of macroautophagy genes in various innate immune cells of KD patients following IVIG therapy. Thus, by inducing autophagy IVIG might counteract inflammasome activation, limit pro-inflammatory cytokines, and correct immune dysregulation, thereby contributing to its therapeutic efficacy.

Beyond bulk macroautophagy, selective autophagy pathways provide cell-type-specific mechanisms for resolving inflammatory stress by removing damaged mitochondria, aggregated proteins, and other cellular cargo (67). Loss of selective autophagy in immune cells is increasingly recognized as a significant factor in the pathogenesis of various inflammatory diseases (67, 72). Reduction in cytotoxicity of NK cells has been reported due to exhaustion and increased damaged mitochondria in inflammatory diseases (73, 74). Studies have shown that failure of mitophagy induces NLRR3 inflammasome activation in monocytes. Thus, defective mitophagy may propagate hyperinflammation in KD through accumulation of damaged mitochondria (41). We found increased mRNA expression of mitophagy related adaptors in monocyte subsets and NK cells following IVIG therapy, indicating that IVIG curbs the inflammation by clearing the damaged mitochondria. Since dysregulated mitophagy is associated with decreased effector functions of NK cells in KD, induction of mitophagy by IVIG might help to improve the number and effector functions of NK cells. Similarly, we also observed increased aggrephagy in γδ T cells after IVIG therapy, which may help in the clearance of protein aggregates accumulated due to excessive inflammation (75). Collectively, these findings support a model in which IVIG does not induce a uniform autophagy response, but instead promotes cell-type-specific selective autophagy programs that collectively contribute to immune recalibration and suppression of hyperinflammation.

Considering the dual role of autophagy pathway as beneficial or detrimental in different pathological settings, therapeutic modulation of autophagy should be tightly regulated. An intermittent rather than constitutive upregulation of autophagy is postulated to be optimally effective and associated with lesser side effects in patients (9, 76). We have carried out additional studies on PBMC to investigate the time kinetics of IVIG-induced autophagy and the possible variations with different sources of IVIG preparations. While the source of IVIG preparation has no consequence on the induction of autophagy, there was a time-dependent increase in the autophagy activity up to 12 hrs. However, no further increase in autophagy was observed in subsequent time point, ruling out a possible risk of excessive induction of autophagy by IVIG at least in an *in vitro* setting.

Data from the various labs have demonstrated that IVIG exerts its anti-inflammatory effects by F(ab’)_2_ or Fc fragments. However, certain mechanisms need entire intact IgG. Our recent data have demonstrated that F(ab’)_2_ fragments of IVIG could induce autophagy in PBMC similar to intact IgG, and the process was independent of sialylation (26). These findings are further supported by current data, wherein either Fc fragments of IVIG or CLRs are found to be not implicated in IVIG-induced autophagy. These data together point towards F(ab’)_2_-dependent modification of signalling events in innate cells leading to induction of diverse autophagy pathways. However, an indirect effect of IVIG on the induction of autophagy in immune cells cannot be ruled out, as IVIG is known to induce changes in the metabolism of immune cells by F(ab’)_2_, Fc fragments and sialylation-dependent manner (37). IVIG promotes the synthesis of various anti-inflammatory lipid molecules like phosphatidylcholine, which is required for the formation of autophagosome membrane and its maintenance during autophagy (77). Phosphatidylethanolamine positively affects the autophagy initiation and maturation by supporting the lipidation of LC3 by attaching LC3 to the autophagosome membrane (78). Furthermore, phosphatidylserine has a role in driving non-canonical autophagy pathway (79). These diverse mechanisms possibly explain at least in part, the induction of various autophagy pathways by IVIG.

The extent to which IVIG affects autophagy in KD patients remains speculative, as direct experimental or clinical evidence in KD is limited. Unlike certain primary immunodeficiency conditions (80), in which intrinsic immune cell defects may render autophagy a central pathogenic pathway, KD is an acute inflammatory vasculitis. In this context, IVIG therapy is more likely to influence autophagy both directly and indirectly through multiple mechanisms, including suppression of pro-inflammatory cytokines, modulation of Fc receptor–mediated signaling, alterations in immune metabolism, and reduction of cellular stress. Therefore, any effect of IVIG on autophagy in KD is expected to be context-dependent and of uncertain magnitude, warranting further investigation.

In summary, in acute KD, impaired macroautophagy and selective autophagy programs contribute to persistent innate immune activation and inflammatory imbalance. IVIG therapy restores autophagy-related homeostatic pathways in a cell-type-specific manner—enhancing macroautophagy and selective programs such as mitophagy (monocytes/NK cells) and aggrephagy (γδ T cells)—thereby limiting inflammasome-driven cytokine amplification, improving immune-cell fitness, and promoting resolution of hyperinflammation.

## Data Availability

The original contributions presented in the study are included in the article/**Supplementary Material**, further inquiries can be directed to the corresponding author/s.
