# Supplementary material for: Intravenous immunoglobulin remodels innate immune cell communication and induces differential autophagy pathways in Kawasaki disease
Source: Front Immunol. 2026 Feb 25;17:1753478. doi: 10.3389/fimmu.2026.1753478 (PMC12975960; doi:10.3389/fimmu.2026.1753478)
Supplement: Supplementary file 1 [file DataSheet1.pdf]

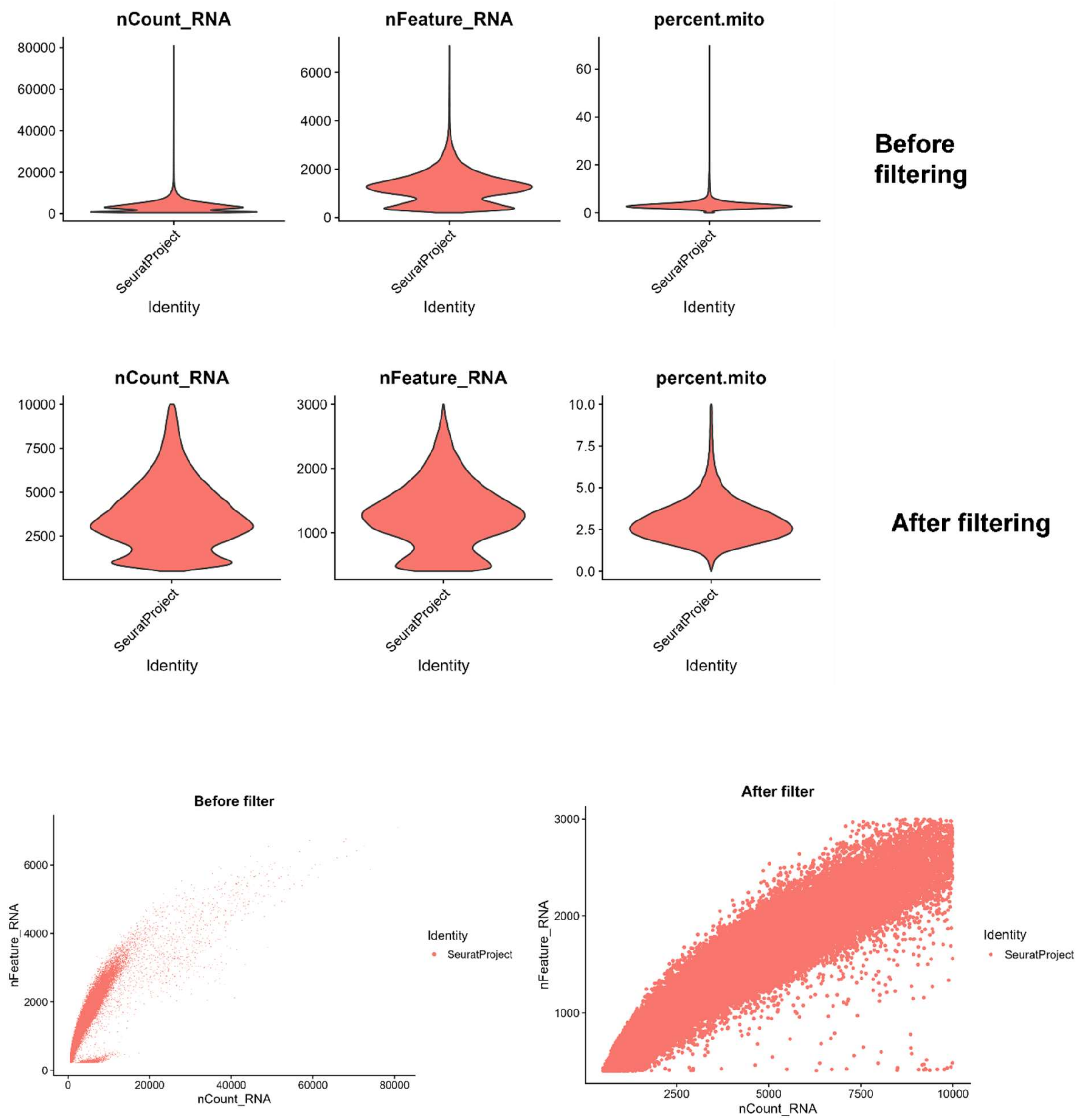

Figure S1. Vlnplots and scatter plots showing counts, features in combined Seurat object before and after filtering.

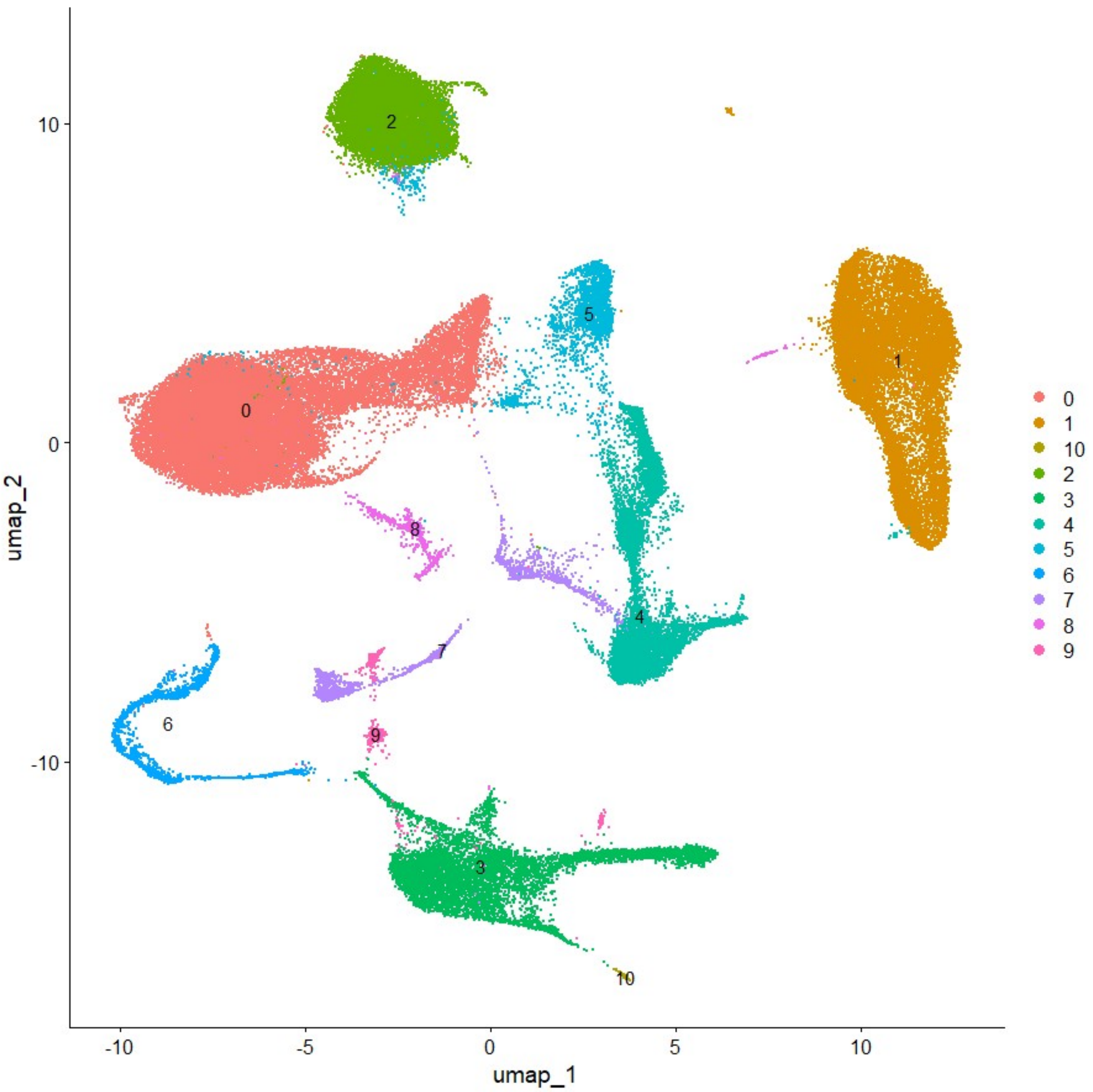

Figure S2. UMAP visualization of the integrated scRNA-seq PBMC dataset showing identified clusters.

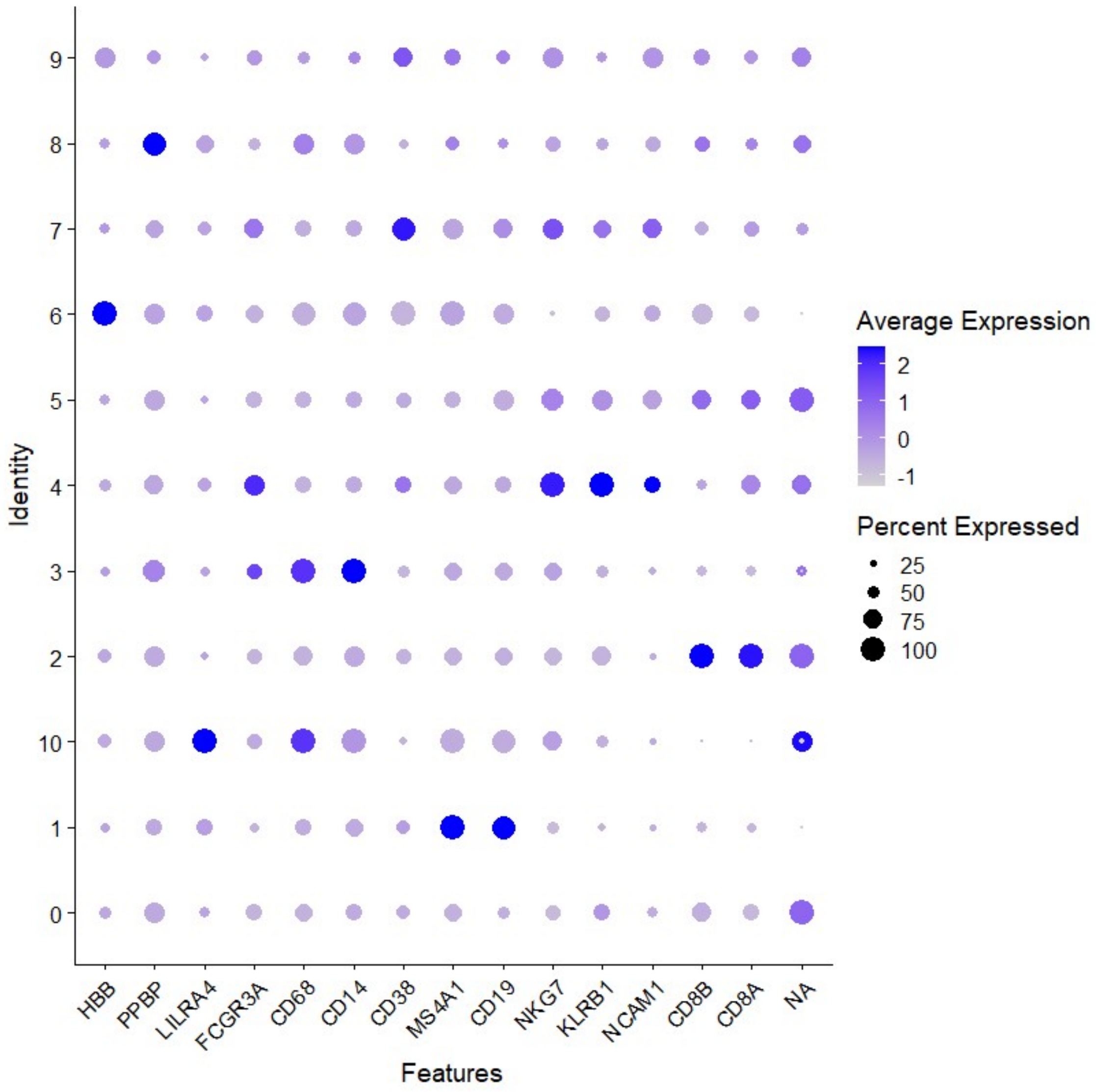

Figure S3. Dot plot of canonical marker gene expression across clusters for PBMC cell-type annotation (color indicates average expression; dot size indicates percent of cells expressing)

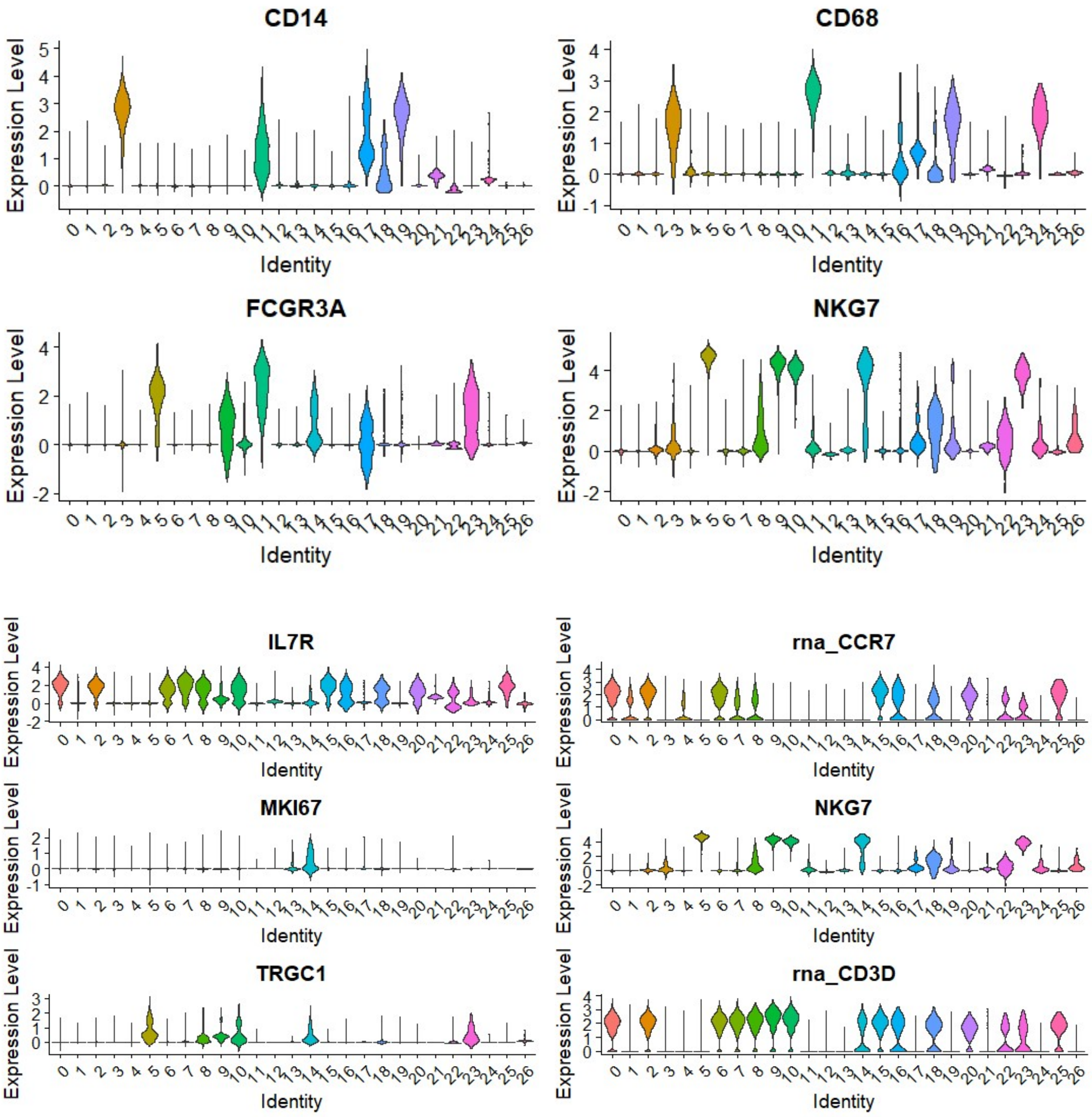

Figure S4. Violin plots of canonical marker gene expression across PBMC clusters used for cell-type annotation.

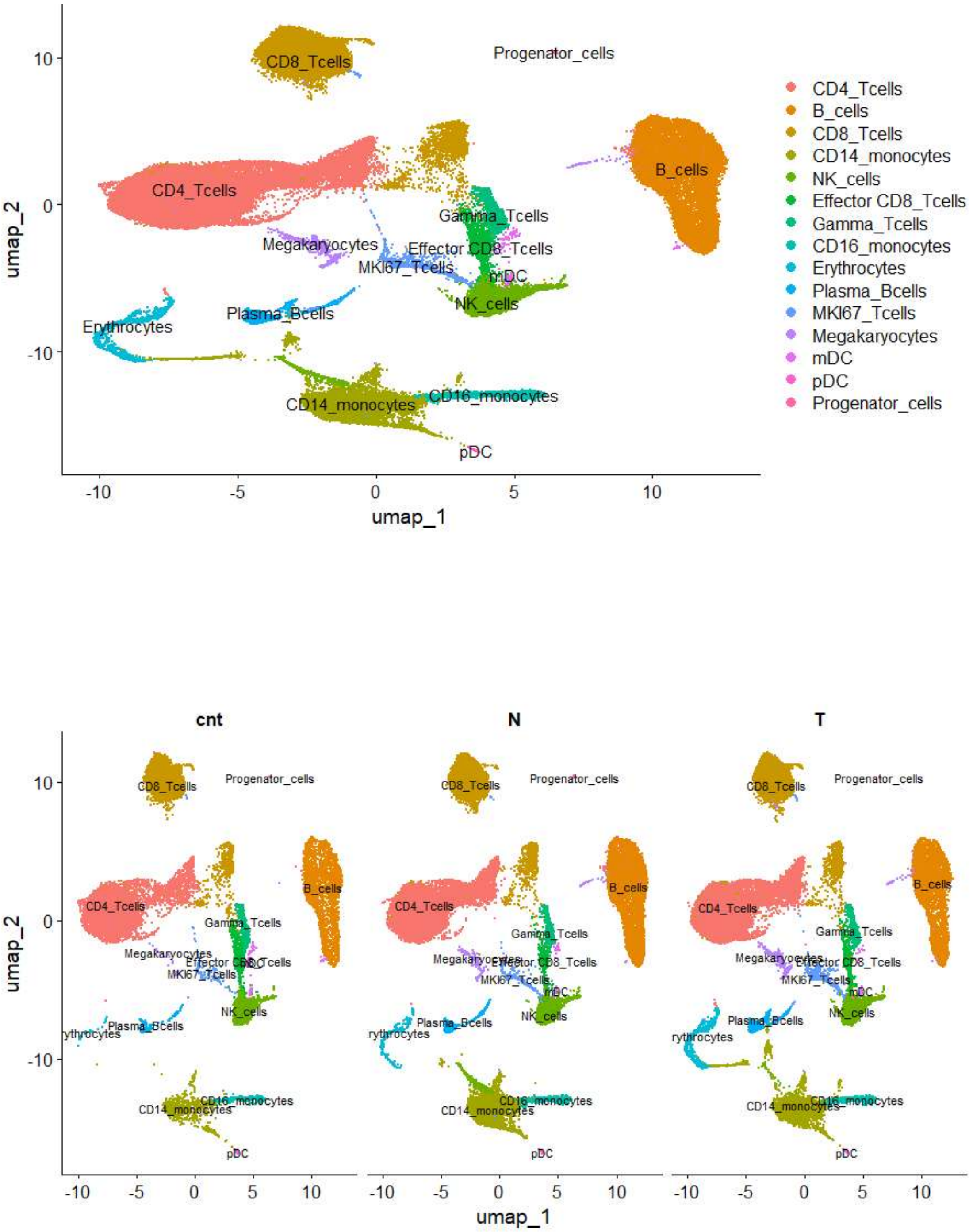

Figure S5. UMAP plots showing annotated PBMC cell types in the integrated dataset and across conditions (control, KD, and post-IVIG).

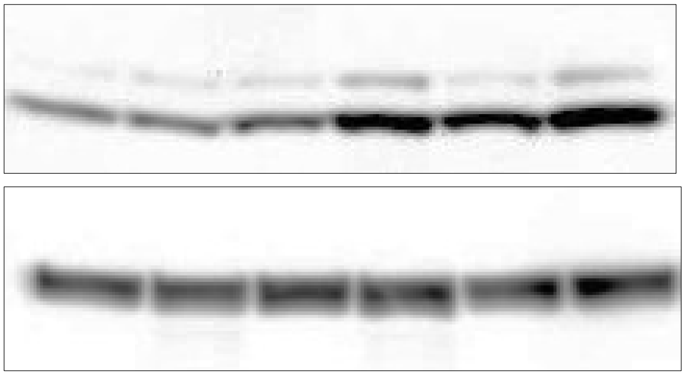

Fig 10

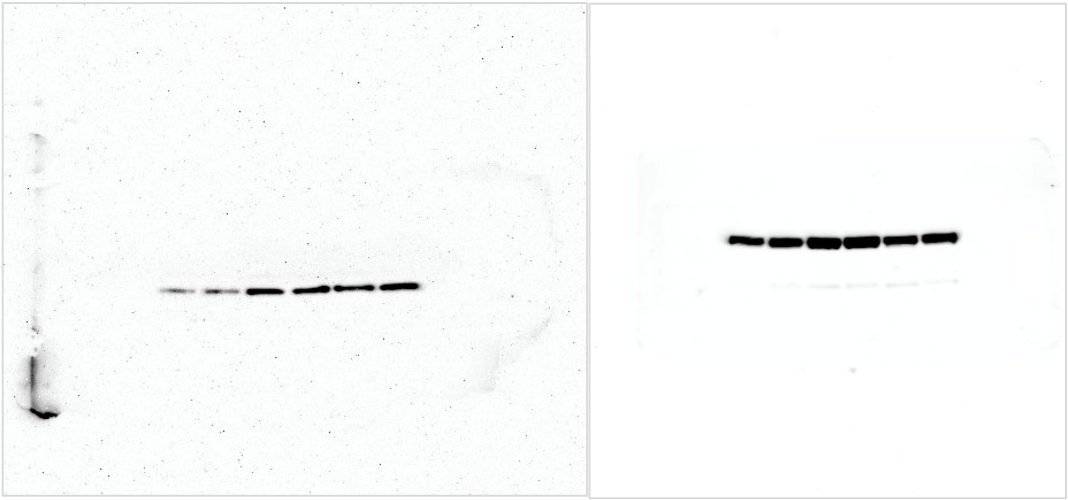

Fig 11

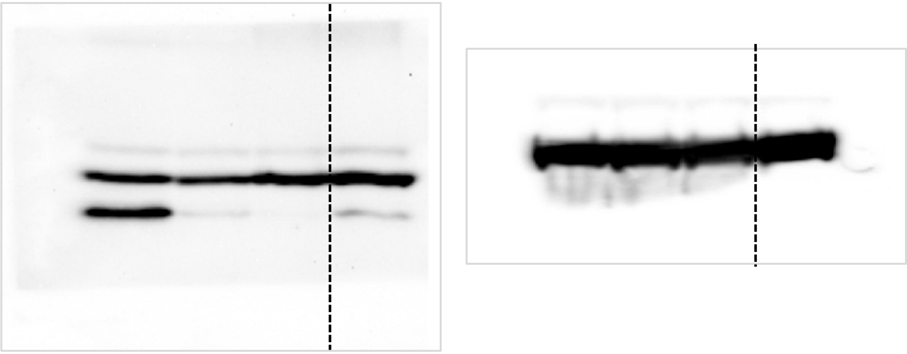

Fig 12 A

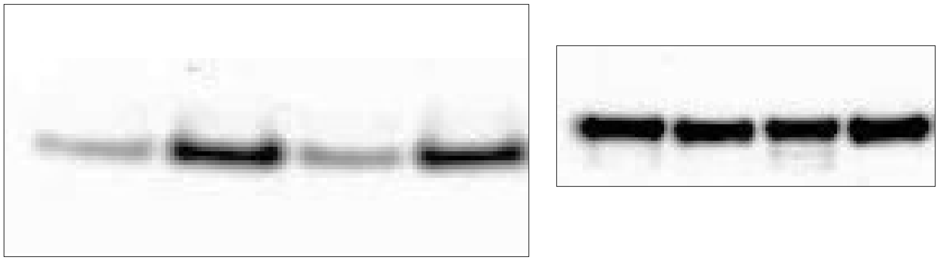

Fig 12 B

Figure S6. Full blots for Figures 10, 11 and 12
